# Incidence of lead dislodgement, malfunction and perforation during the first year following device implantation

**DOI:** 10.1007/s12471-014-0556-6

**Published:** 2014-05-08

**Authors:** A. Ghani, P. P. H. M. Delnoy, A. R. Ramdat Misier, J. J. J. Smit, A. Adiyaman, J. P. Ottervanger, A. Elvan

**Affiliations:** Department of Cardiology, Isala Hospital, Dr. Van Heesweg 2, 8025 AB Zwolle, the Netherlands

**Keywords:** Lead complication, Dislodgement, Malfunction, Perforation, Re-intervention

## Abstract

**Background:**

The number of cardiac rhythm device implantations has been growing fast due to expanding indications and ageing of the population. Complications of implantation were rare in the trials. However, these involved small numbers and selected patients. Prospective real-life data are necessary to assess cardiac device implantation procedure-related risks.

**Objective:**

To determine the incidence and predictors of lead-related re-intervention in a Dutch high-volume teaching hospital.

**Methods:**

Data from all patients who underwent cardiac rhythm device implantation between January 2010 and December 2011 were collected in a prospective registry. At least 1 year of follow-up regarding re-intervention was available for all patients. Lead-related reasons for re-intervention were categorised into lead dislodgement, malfunctioning or perforation.

**Results:**

One thousand nine hundred twenty-nine devices including 3909 leads were implanted. In 595 patients (30.8 %) a CRT-D/P was implanted. Lead-related re-intervention was necessary in 86 (4.4 %) patients; it was more common in younger and male patients, and due to either lead dislodgement (66 %), malfunctioning (20 %) or perforation (18 %). Coronary sinus lead dislodgement or malfunctioning was 1.4 %. Right atrial dislodgement (1.9 %, *p* < 0.001) or ICD lead dislodgement (1.8 %, *p* = 0.002) was more common than right ventricular dislodgement (0.3 %). The incidence of lead malfunctioning was higher (0.8 %) in ICD leads. An apical position of the right ventricular lead and lateral wall position of the right atrial lead were related to cardiac perforation.

**Conclusions:**

The incidence of lead-related re-intervention was comparable with the literature. The majority of re-interventions were due to lead dislodgements, particularly with right atrial and ICD leads. Re-intervention due to coronary sinus lead dislodgement was rare.

## Introduction

The number of cardiac rhythm device implantations, including implantable cardioverter defibrillator (ICD) and cardiac resynchronisation therapy (CRT) devices, has increased fast in the past decade due to expanding indications and ageing of the population. Although the benefits of these devices were demonstrated in randomised controlled trials, this concerned selected patients and real-life data are necessary to assess cardiac device implantation-related risks. Several prospective and retrospective studies reported both short- and long-term complications related to device implantation and pacing system upgrade. However, the majority of these reports are derived from randomised clinical trials which reflect selected patients and circumstances [1] whereas other studies concern relatively old reports, also including leads with passive fixation, which are nowadays less commonly used [2–8].

The objective of this prospective registry study was to assess the real-life incidence of lead dislodgement, malfunctioning or perforation during the first year following implantation in a Dutch high-volume teaching hospital.

## Methods

Data on all patients who underwent procedures of de novo cardiac rhythm device implantations and pacing system upgrades in our hospital between January 2010 and December 2011 were prospectively collected. The indications for the implantation of pacemakers, ICD and CRT devices were based on contemporary guidelines [9, 10]. The procedures were performed by seven operators including two cardiac electrophysiology fellows under direct supervision of an attending cardiac electrophysiologist, in cardiac catheterisation laboratories equipped according to the guidelines of the European Society of Cardiology (9). The leads and devices were implanted according to the manufacturers’ recommendations. The baseline characteristics of patients including age, gender, left ventricular ejection fraction (LVEF), presence of conduction disorders and functional New York Heart Association (NYHA) class were recorded in the prospective database. To identify the lead-related complications the database was searched for re-intervention procedures during the first year following the implantation. In all lead-related re-intervention cases, data on the clinical manifestation and course, results on chest X-ray, echocardiography and technical data on lead performance were collected.

## Definition of complications

**Lead dislodgement** was defined if there was documentation of a change in the lead tip position on chest X-ray and changes in electrical lead parameters (rise in impedance, loss of sensing and pacing).

**Lead electrical malfunctioning** was defined if lead impedance, electrogram amplitude or threshold had changed abruptly necessitating surgical revision without clear changes in the position of the lead on chest X-ray.

**Lead perforation** was defined in case of high suspicion of cardiac perforation, e.g. an acute stabbing chest pain or dyspnoea, significant changes in electrical lead parameters and a significant amount of pericardial effusion requiring pericardiocentesis with or without extracardiac lead location on X-ray.

**Screw perforation** was defined if there were pericarditis-like symptoms without clear changes in electrical lead parameters and absence of a significant amount of pericardial effusion.

### Statistical analysis

Statistical analysis was performed using SPSS statistical software (IBM Corp. Released 2011. IBM SPSS Statistics for Windows, Version 20.0. Armonk, NY: IBM Corp). Continuous variables are expressed as mean ± SD and P value was calculated by using the ANOVA test. Categorical variables were presented as numbers and percentages and significance of differences were analysed using the *χ* ^2^ test or Fisher’s exact test. The denoted p values were two-sided and *p* < 0.05 was considered significant.

## Results

**Fig. 1:**
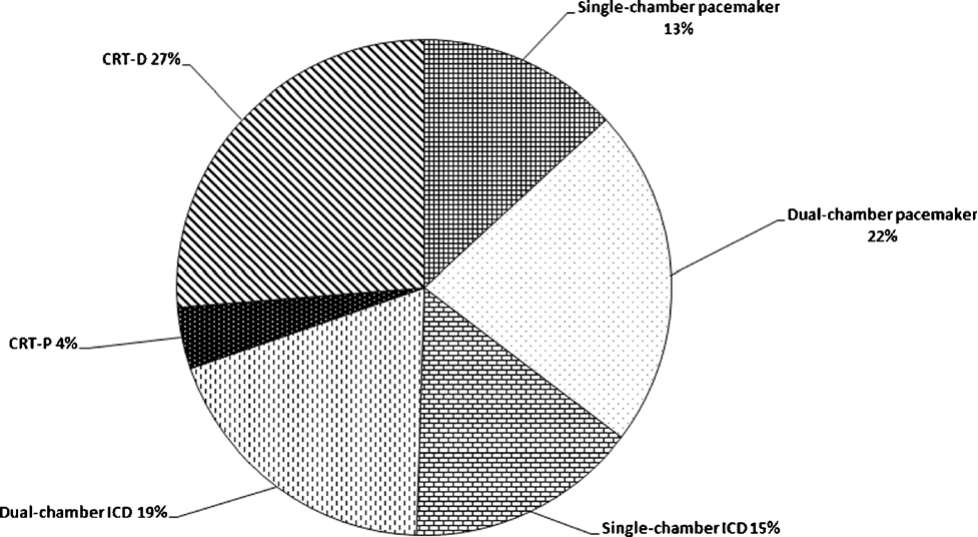
A total of 1929 cardiac rhythm devices implanted

**Fig. 2:**
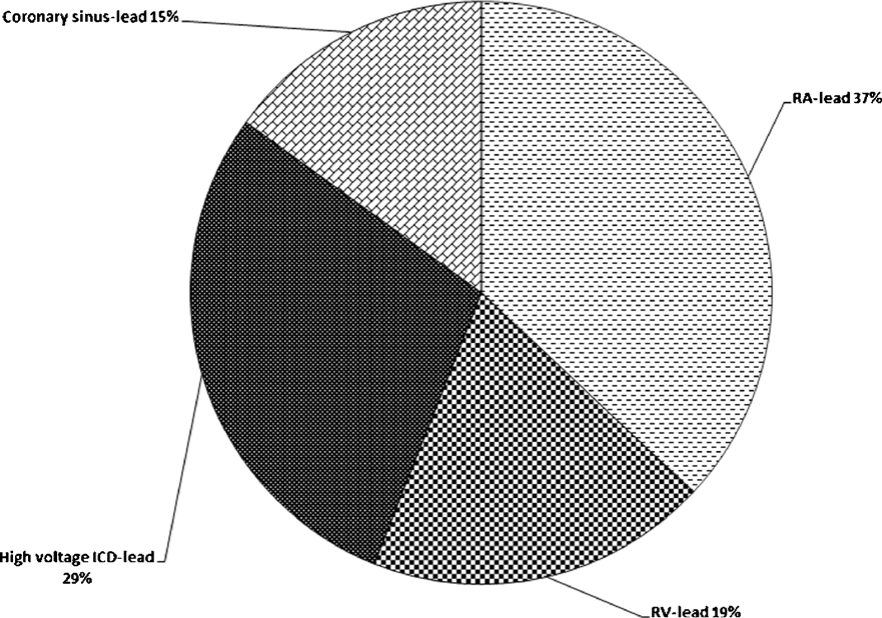
A total of 3909 leads implanted

During a period of 2 years, 1929 cardiac rhythm devices and 3909 leads were implanted in 1929 consecutive patients with commonly accepted indications for either pacemaker, ICD or CRT device implantation. The baseline characteristics of patients and type of device implantation are summarised in Table 1. Patients with pacemaker indications were older than ICD patients and 66 % of patients with cardiac rhythm device implantations were male. All leads except the coronary sinus leads were actively fixed. Device and lead manufacturers included Medtronic, St. Jude Medical, Biotronik, Sorin Group and Boston Scientific. Of all implanted devices, 1148 (60 %) were ICDs and 1148 (29 %) of all implanted leads were ICD leads. Details regarding implanted devices and leads are summarised in Figs. 1 and 2.

**Table 1:** Clinical characteristics

Baseline	Single-chamber PM *N* = 259	Dual-chamber PM *N* = 426	Single chamber ICD *N* = 287	Dual chamber ICD *N* = 364	CRT-P *N* = 91	CRT-D *N* = 504	*P*-value
Age (year)	78 ± 11	74 ± 11	61 ± 13	65 ± 11	72 ± 12	69 ± 9	*P* < 0.001
Male (%)	55	55	72.5	77	65	76	*P* < 0.001
LVEF (%)	–	–	30 ± 9	31 ± 12	40 ± 13	26 ± 11	*P* < 0.001
NYHA class	–	–	2.1 ± 0.5	2.2 ± 0.6	2.3 ± 0.9	2.4 ± 1	*P* = 0.1
QRS duration (ms)	–	–	112 ± 33	112 ± 40	143 ± 60	161 ± 59	*P* < 0.001

P value is calculated by using ANOVA test

*CRT* cardiac resynchronisation therapy, *ICD* implantable cardioverter defibrillator, *LVEF* left ventricular ejection fraction, *NYHA class* New York Heart Association functional class, *PM* pacemaker

Thirty-one (1.5 %) patients were re-admitted because of cardiac device infection, and all had explantation of their device. One (3.2 %) patient died within 30 days of hospitalisation. Positive cultures were present in 27/31 (87 %) cases. These consisted predominantly of micro-organisms that are part of the skin flora (84 %).

### Re-intervention

A total of 90 (2.3 % of the leads) lead-related complications occurred in 86 (4.4 %) patients for which re-intervention was needed. The cause of re-intervention was mainly lead dislodgement (66 %), followed by malfunctioning (20 %) and perforation (18 %). Re-intervention was more common in men compared with women (70 % vs 30 % *p* = 0.001). Younger patients more often had re-intervention (mean age 65 ± 13 vs 70 ± 13 years, *p* = 0.001).

### Lead dislodgement or malfunction and time of occurrence

**Fig. 3:**
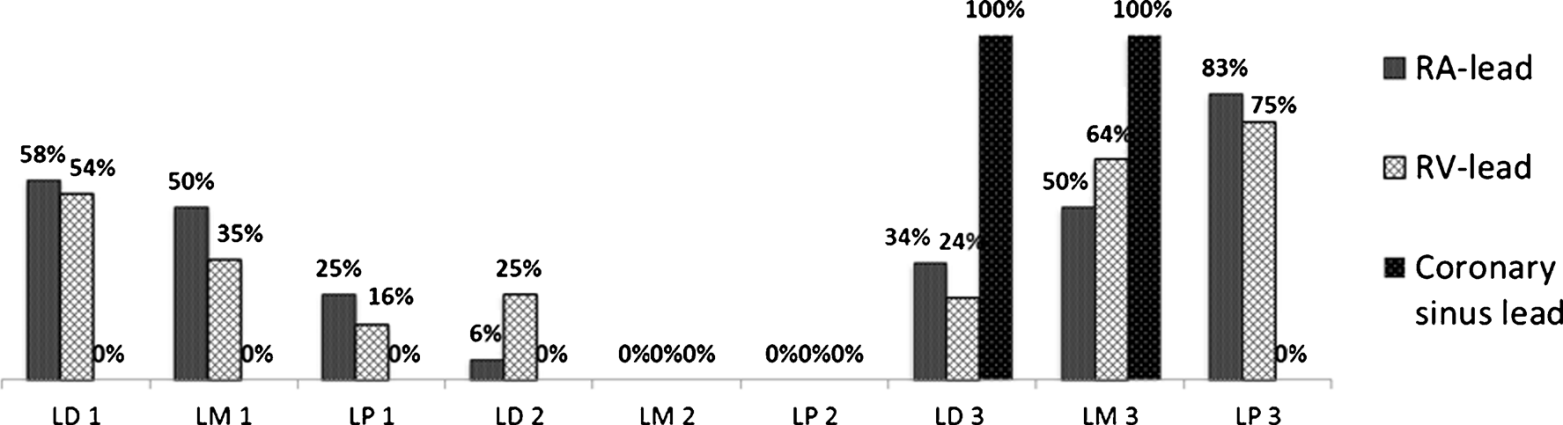
Timing of either lead dislodgement, malfunctioning or perforation. The majority of lead dislodgements occurred before discharge and all perforations occurred after the 2nd month of implantation. *LD 1* lead dislodgement before discharge, *LM 1* lead malfunctioning before discharge, *LP 1* lead perforation before discharge, *LD 2* lead dislodgement between discharge and 2 months, *LM 2* lead malfunctioning between discharge and 2 months, *LP 2* lead perforation between discharge and 2 months, *LD 3* lead dislodgement between 2 months and 1 year, *LM 3* lead malfunctioning between 2 months and 1 year, *LP 3* lead perforation between 2 months and 1 year

A total of 3909 leads were implanted. During the first year of follow-up a total of 74 (1.9 %) lead dislodgements or malfunctions occurred in 71 (3.7 %) patients: 57 (1.4 %) dislodgements and 17 (0.5 %) malfunctions. Regarding lead dislodgement, the right atrial (RA) lead (1.9 %) showed the most frequent lead dislodgement compared with the right ventricular (RV) pacemaker lead (0.3 %) or ICD lead (1.8 %) (*p* = 0.0007 and *p* = 0.002), Table 2. Only 6 (1 %) coronary sinus (CS) leads dislocated requiring re-intervention. Regarding lead malfunction, the ICD lead (0.8 %) showed the most frequent lead malfunction compared with the RA lead (0.1 %) (*p* = 0.002), Table 2. The timing of occurrence of lead dislodgement/malfunction is summarised in Fig. 3. The majority of RA and RV lead dislodgements occurred before discharge whereas the majority of RA and RV lead malfunction occurred after the 2nd month following implantation. All CS lead dislodgements or malfunctions occurred after the 2nd month following implantation. In almost 1/3 of the cases the sleeves on the leads were not fixed adequately and in 2/3 of the cases the cause of dislodgement was unclear. All re-do interventions were conducted without further complications.

**Table 2:** Lead dislodgement, malfunctioning and perforation

Type of lead	Number implanted	Dislodgement (%)	Malfunctioning (%)	Lead perforation (%) with pericardiocentesis	Screw perforation (%) without pericardiocentesis
Atrial leads	1442	28 (1.9)^Ɨ^	2 (0.1)	5 (0.3)	2 (0.1)
RV pacemaker leads	724	2 (0.3)	4 (0.5)	1 (0.1)	7 (0.9)
ICD leads	1148	21 (1.8)^ǂ^	9 (0.8)	–	1 (0.08)
Coronary sinus leads	595	6 (1)^£^	2 (0.3)	–	–
Total	3909	57 (1.5)	17 (0.4)	6 (0.15)	10 (0.25)

P value is calculated by using Fisher exact test

*ICD* implantable cardioverter defibrillator, *RV* right ventricular

^Ɨ^ *P* = 0.0007 compared with RV pacemaker lead as reference

^ǂ^ *p* = 0.002 compared with RV pacemaker lead as reference

^£^ *p* = 0.15 compared with RV pacemaker lead as reference

### Lead dislodgement per device type

In this study 595 CRT-D/P devices were implanted. In the CRT-D group, significantly more lead dislodgments or malfunctions were observed compared with the single-chamber pacemaker (30 versus 3, *p* = 0.006). The risk of any lead dislodgement or malfunctioning was higher in CRT-D (5 %, *p* = 0.006) and dual-chamber ICD (5.8 %, *p* = 0.002) as compared with single-chamber pacemaker (1.2 %). Numbers of lead dislodgements per type of device are summarised in Table 3.

**Table 3:** Lead dislodgement and type of device

Type of device	Number of lead dislodgement or malfunction	Risk of any leads lead dislodgement or malfunction	Number of lead/screw perforation with or without pericardiocentesis	Risk of lead/screw perforation with or without pericardiocentesis
Single chamber PM	3	1.2 %	3^*^	0.8 %
Dual chamber PM	10	2.3 %	12^#^	3.0 %
Single chamber ICD	10^†^	3.5 %	1	0.3 %
Dual chamber ICD	21^ǂ^	5.8 %	0	0 %
CRT-D/P	30^£^	5.0 %	0	0 %

*CRT* cardiac resynchronisation therapy, *ICD* implantable cardioverter defibrillator, *PM* pacemaker

^†^ *p* = 0.09 compared with single chamber pacemaker as reference

^ǂ^ *p* = 0.002 compared with single chamber pacemaker as reference

^£^ *p* = 0.006 compared with single chamber pacemaker as reference

^*^ *p* = 0.35 compared with single chamber ICD as reference

^#^ *p* = 0.02 compared with single chamber ICD as reference

### Lead or screw perforation

During follow-up, 16 leads (0.4 % of the leads) showed screw/lead perforation in 15 (0.8 %) patients. They were re-admitted to the hospital with pericarditis-like symptoms suspected for cardiac perforation. The clinical presentation of all suspected cardiac perforations was subacute (±21 days after implantation). Fourteen patients were implanted with single- or dual-chamber pacemaker and one patient with single-chamber ICD. The majority of these patients were re-admitted after the first week of implantation (Fig. 3). Ten patients were re-admitted with pericarditis-like symptoms with a slight amount (<0.5 cm) of pericardial effusion on the echocardiogram. In these patients only repositioning of the lead was sufficient without need for pericardiocentesis. The other five patients were re-admitted with symptoms of cardiac tamponade and a significant amount of pericardial effusion (>2 cm, with >25 % respiratory mitral flow variation) on the echocardiogram, suspect for lead perforation without obvious extracardiac location of a lead on chest X-ray. In these 5 (0.26 %) patients, with dual-chamber pacemakers, pericardiocentesis was necessary and performed. In all cases a repositioning or implantation of a new lead was performed without further complications and without the need for thoracic surgical intervention. In all patients, the pericarditis-like symptoms disappeared after repositioning of the suspected lead. From the 16 (0.4 %) lead/screw perforations, 7 were RA leads, 8 were RV pacemaker leads and 1 was an active fixation ICD lead. RV pacemaker leads caused significantly more lead/screw perforation compared with ICD leads (*p* = 0.02), Table 3. All RV leads causing perforations were located in RV apex region and four out seven RA leads which caused perforation were located in the lateral wall of the right atrium.

### Lead or screw perforation per type of device

Most lead/screw perforations (12 cases) occurred in dual-chamber pacemakers compared with single-chamber pacemakers (2 cases) or single-chamber ICD (1 case) (*p* = 0.02). There were no lead/screw perforations in dual-chamber ICD or CRT-D/P devices. The rate for lead/screw perforation in dual-chamber pacemakers was 3 %, whereas this did not occur in CRT-D/P patients. Numbers of leads/screw perforations per type of device are summarised in Table 3.

## Discussion

In this prospective device complication registry of 1929 patients, 4.4 % of patients suffered from a lead dislodgement, malfunctioning or perforation during the first year following the implantation. The overall rate of lead dislodgement, malfunctioning or perforation requiring re-intervention was low (2.3 % of 3909 leads) in our study. RA and ICD leads were the leads with the highest risk (1.9 %) of dislodgement compared with RV pacemaker and coronary sinus leads. The rate of lead malfunctioning was higher in ICD leads. The rate of coronary sinus lead dislodgement or malfunction within the first year following the implantation was very low in this study (1.4 %). Dual-chamber ICD implantation was the procedure with the highest risk of lead dislodgement and DDD pacemaker implantation with the highest risk of lead perforation. The overall lead dislodgement rate in our hospital is low and comparable (1.5–3.3 %) with published studies [2, 6, 10–14]. The CRT trials [2, 13–15] reported any lead dislodgement rate varying from 2.9 to 10.6 %. In our hospital the rate of any lead dislodgement in CRT-D/P devices was 5 %. In CRT devices the lead failure rate exceeds the rate of lead failure in one- or two-chamber devices. This is comprehensible since more leads are implanted per device. The low rate of any lead dislodgements of 5 % in our group could be explained by a low rate of coronary sinus lead dislodgements. The rate of coronary sinus lead dislodgement in the present study is low (1.4 %) compared with previous reports (4.0–8.4 %) [16, 17]. There are several possible explanations. The coronary sinus leads are today more diverse with different shapes and thicknesses. These properties enable the operators to choose the lead which fits best in the side branches of the coronary sinus. Also the use of inner catheters and selective shackling of the side branches makes it easier to advance the lead inside the vessel and to achieve a stable position. A possible other explanation is the experience of the operator. Almost all coronary sinus leads were implanted by three very experienced operators in our hospital. Furthermore, since coronary sinus leads of all different manufactures are available in our hospital during the implantation, and there is no preferred manufacturer assigned to a case, the most suitable lead can be chosen to be implanted according to the anatomy, enhancing the success rate. We tried to identify the causes of lead dislodgments. In almost 1/3 of the cases the sleeves on the leads were not fixed adequately and in 2/3 of the cases the cause of dislodgement was unclear. This means that in 1/3 of the cases a lead dislocation could be prevented by adequate fixation. In the present study all lead/screw perforations were subacute and the incidence was very low (0.8 %) and comparable with (0.6–5.2 %) the published reports [1, 5, 7]. DDD pacemakers caused the most perforations (12 cases) and the majority of perforated leads were RV leads. There are several possible explanations. 1) All perforated RV leads were located in the RV apex which is the thinnest part of the right ventricle. In our routine practice we try to avoid the apex if possible and position the RV leads in a septal region if sufficient sensing and pacing values are present. 2) The relatively small size of the lead, 6–7 French, which more easily perforates the apex compared with the thick high-voltage ICD (8–9 French) lead and, according *to Laplace’s law* (p = F/A; pressure is the amount of force acting on a unit area), it is easy to understand that when the surface area is smaller, then the pressure will be higher on that surface area. 3) Also the experience of operators plays an important role. In our teaching hospital most pacemakers were implanted by less experienced operators, cardiologists in training, and ICDs and CRTs by very experienced operators. The majority of perforated RA leads were located in the lateral wall, which is not the preferred site, due to unstable position or insufficient electrical signals and therefore the operator was forced to choose for this site.

## Strength and limitations of study

This study demonstrates the real-world common daily practice by implanting a large number of devices in a teaching hospital. Meticulous longitudinal follow-up was performed, with documentation of complications in all patients. This study also has certain limitations. We identified the re-intervention due to lead-related complication when the patients were returned to cardiac catheterisation laboratories within first year of implantation. Patients with lead-related complications who did not return for re-intervention based on the opinion of their cardiologists could be missed in our registry. Furthermore, the follow-up period in the present study is no longer than 1 year. However, most lead dislocations and perforations are expected to occur within 1 year [1, 7, 11]. This is also supported by our own data. To obtain reliable information about the rate of complications, we used predefined definitions of complications. We focused on lead dislodgement, malfunctioning and perforations. Other device-related complications, including infection, were beyond the scope of this study. Our analysis also did not evaluate mortality and duration of hospitalisation in the study population.

## Conclusions

In this large observational study, lead related re-intervention was necessary in 4.4 % of patients, more common in younger and male patients, and due to either lead dislodgement (66 %), malfunctioning (20 %) or perforation (18 %). With a total of 3909 leads implanted, the incidence of lead dislodgement, malfunction or perforation was low (2.3 %). Right atrial and ICD leads caused more dislodgement compared with RV pacemaker leads. Lead perforation was more common with RV leads, especially when placed in the apex. In the patients with lead perforation, pericardiocentesis was only necessary in 1/3. With 1929 devices implanted, more lead complications were observed in dual-chamber ICD and CRT-D/P.
